# Single Leg Drop and Hop: Insight Into Multisegment Foot Kinematics, Kinetics and the Role of Visual Focus in Healthy Young Adult Males

**DOI:** 10.1002/jfa2.70078

**Published:** 2025-09-01

**Authors:** Nicolas Haelewijn, Filip Staes, Evie Vereecke, Stijn Rosseel, Kevin Deschamps

**Affiliations:** ^1^ Department of Rehabilitation Sciences Musculoskeletal Rehabilitation Research Group KU Leuven Brugge Belgium; ^2^ Department of Rehabilitation Sciences Musculoskeletal Rehabilitation Research Group KU Leuven Leuven Belgium; ^3^ Department of Development & Regeneration Campus Kulak KU Leuven Kortrijk Belgium

**Keywords:** joint power, motor control, proprioception, visual input

## Abstract

**Introduction:**

Understanding foot joint loading during different dynamic activities is essential information for guiding exercise progression in rehabilitation. While walking and running biomechanics are well studied, joint‐specific kinetic data during a single leg drop and hop task, often used in rehabilitation, are lacking. This study aimed to evaluate (1) the kinetic behavior of the ankle, Chopart, Lisfranc, and MTP‐1 joints during a drop‐hop task under different visual constraints and (2) to contextualize these findings by comparing them with heel‐strike running, to assess the relative loading demands of the drop‐hop task.

**Methods:**

Seventeen recreationally active male adults performed a single‐leg drop and hop under two visual focus conditions: central (focusing on the landing spot) and peripheral (focusing straight ahead). Kinematics, moments, and power were analyzed using a four‐segment foot model with statistical parametric mapping. Additionally, peak plantarflexion moments and power outputs were compared with existing data from heel‐strike running data from a mixed‐sex sample (4 males, 3 females) collected in a separate study using the same setup.

**Results:**

Findings revealed no differences between central and peripheral focus conditions. Heel‐strike running shows similar joint loading, but higher power generation (*p* < 0.001) at the ankle and Chopart joint, higher absorption (*p* < 0.001) at the Chopart and MTP‐1 (*p* < 0.05) joint and lower power absorption (*p* < 0.001) at the ankle and Lisfranc joint.

**Conclusion:**

Visual input does not influence foot biomechanics during a single‐leg drop and hop. This task produces similar joint loading patterns similar to heel‐strike running but with reduced power generation at the ankle and midfoot. Contrary to global belief, the single leg drop‐hop task is not excessively more demanding in terms of foot joint loading, supporting the earlier use of drop‐hop exercises in rehabilitation programs. They offer a controlled way to reintroduce loading while avoiding the full propulsion demands of running, independent of visual focus.

## 1 Introduction

Successful rehabilitation focuses on restoring function while ensuring a safe and gradual progression of exercise intensity and complexity [1]. Two primary approaches are commonly used to guide the progression of exercise loads [2]. The first approach is time‐based, where loading progression is solely determined by the time elapsed since injury [3]. This approach is easy to follow as it aligns with the general time frames of tissue healing, including hemostasis, inflammation, proliferation, and remodeling. However, it overlooks the actual readiness of the tissue, which may lead to disorganized scar formation, joint stiffness, and muscle atrophy, ultimately delaying recovery [3]. In contrast, the performance‐driven approach is more individualized, advancing only when specific criteria are met [4, 5]. This allows for controlled loading, promoting tissue healing and improving mechanical properties.

Biomechanics plays a key role in guiding exercise progression. Over the past decade, significant progress has been made in understanding joint loading and exercise progression, particularly for the groin [6, 7, 8] and knee region [9, 10]. These advancements have refined rehabilitation protocols, particularly during the advanced stages when multi‐joint exercises are emphasized [7]. However, knowledge on progression at the level of the foot remains limited, as literature on joint loading is scarce, despite the foot serving as the primary interface between the body and the ground. Understanding joint loading at the foot level can help reduce the risk of stress fractures, plantar fasciopathy, and tendon overuse injuries [11].

Single‐leg exercises such as single‐leg drops and hops are commonly incorporated into rehabilitation and strength training programs. However, previous research on foot joint loading has primarily focused on walking and running biomechanics. For example, Deschamps et al. (2020) reported peak plantarflexion moments at the ankle (2.9 Nm/kg), Chopart (2.16 Nm/kg), Lisfranc (1.04 Nm/kg), and MTP‐1 (0.27 Nm/kg) joints during running [12]. These findings do however not directly translate to more functional tasks such as single‐leg drops and hops. Only Kessler et al. (2020) have investigated how foot quasi‐stiffness is regulated during hopping across a range of frequencies, demonstrating that midfoot quasi‐stiffness increases with hopping frequency [13]. However, quasi‐stiffness reflects a combined effect of muscle‐tendon behavior and joint moment‐angle relations, and does not reveal the intersegmental joint kinetics underlying foot function. Given the higher impact forces and dynamic stability demands of hopping compared to walking and running [14, 15], it is essential to examine how the different foot joints distribute load and perform mechanical work.

Not only biomechanics are important in task performance. Also the interplay between the different body systems, is important, such as the sensory input, for example vision [16, 17]. The visual system provides critical information for spatial awareness, postural control, and movement planning [18, 19]. Athletes and patients often perform landing and hopping tasks under visual constraints, such as tracking an opponent or focusing on external cues in rehabilitation. More importantly, visual focus can be actively controlled, shifting between central focus (looking directly at the landing spot) and peripheral focus (looking straight ahead or at another object) [20]. Although peripheral focus is often used in dynamic sports situations, its effect on landing mechanics at the foot level remains largely unexplored. Only two studies have investigated visual focus effects on single‐leg landings [20, 21]. Terada & Gribble (2015) reported reduced ankle eversion moments at initial contact, but provided only descriptive data. Ko et al. (2022) found increased ankle inversion and hip adduction during the landing phase in peripheral focus conditions, though effect sizes were small (Cohen's *d* < 0.35) and propulsion was not considered. Given the importance of both power absorption (eccentric phase) and power generation (concentric phase) in rehabilitation exercises, a comprehensive biomechanical analysis across the entire landing cycle is warranted.

Although most prior studies investigating visual feedback during landing tasks have focused on joint kinematics, particularly at the ankle and hip, less is known about how visual constraints may influence joint kinetics. Visual input is a key component of sensorimotor integration, particularly for tasks involving precision landings [17]. Theoretical frameworks suggest that alterations in visual input can lead to changes in neuromuscular coordination and loading strategies to preserve stability under uncertainty. Given the foot's complex multisegmental architecture and its critical role in absorbing and redistributing ground reaction forces, it is plausible that visual constraints could elicit subtle changes in foot joint kinetics even in the absence of larger kinematic adaptations.

By investigating the in vivo foot joint biomechanics and the role of visual input during demanding tasks such as single‐leg drops and hops, trainers and clinicians can gain insight into how these tasks are performed in asymptomatic individuals. Establishing these baseline movement patterns is essential, as they provide a reference for normal foot joint function. This study aims to compare 3D multi‐segment foot biomechanics of the ankle, Chopart, Lisfranc and MTP‐1 joint for single‐leg drop and hop between two conditions in asymptomatic subjects: one with a central visual focus and the other with a peripheral visual focus. Given the complex multisegmental nature of the foot and its role in fine‐tuned adjustments during landing, we hypothesize that subtle kinetic adaptations might emerge under altered visual input. Specifically, we hypothesized that the peripheral visual focus will exhibit higher power absorption, as compensatory adjustments may be needed to maintain balance. A direct comparison with heel‐strike running allows us to contextualize foot joint loading within a well‐documented locomotor task [12]. Identifying similarities and differences between running and hopping, may help provide a biomechanical basis for designing load strategies based on task specificity in rehabilitation.

## 2 Materials and Methods

### 2.1 Participant Details

This study received ethical approval (MP019006) from the University Hospitals Leuven, and written informed consent was obtained from all participants. Seventeen asymptomatic, recreationally active male adults (age 23.1 ± 0.6 years) were recruited through campus advertisements (Table 1). Although sex was not an eligibility criterion, all recruited participants happened to be male. Physical activity levels were assessed using the Short Form International Physical Activity Questionnaire (SF‐IPAQ), with participants required to achieve at least a moderate activity level [22] Exclusion criteria included lower limb injuries or surgery in the past 6 months, recent ankle or foot pain, significant foot deformities, neurological or systemic conditions, or pregnancy. Foot posture was clinically evaluated using the Foot Posture Index‐6 (FPI‐6) [23]. Results on foot joint loading in a running task from Deschamps et al. (2020), were used to contextualize results from the drop and hop [12]. The running study was conducted in a different laboratory within the same university, using the same laboratory setup and data collection procedures. The study included seven asymptomatic adults (4 males, 3 females, age 24.0 ± 4.2 years) who performed barefoot heel‐strike running at an average speed of 3.5 m/s along a 10‐m walkway. Although the sex distribution differed between both studies, no significant differences were observed in other demographic characteristics such as age, height, mass, and BMI. This comparative analysis is intended to provide contextual biomechanical insight into foot joint kinetics across functional tasks, rather than serve as a formal between‐group comparison.

**TABLE 1:** Demographic data of the study cohort (mean and standard deviation).

	This study	Deschamps 2020
	*N* = 17	*N* = 7
Male/female	17/0	4/3
Age (years)	23.1 (0.6)	24.0 (4.2)
Height (m)	1.8 (0.1)	1.7 (0.1)
Mass (kg)	75.6 (8.5)	70.2 (8.9)
BMI (kg/m^2^)	24.0 (2.8)	22.7 (1.9)
Running speed (m/s)		3.5 (0.34)
FPI	3.0 (2.7)	—
Shoe size	43.3 (1.3)	—
SF‐IPAQ (met/min/week)	45,671 (2150.9)	—

Abbreviations: BMI, body mass index; FPI‐6, foot posture index; SF‐IPAQ, short form international physical activity questionnaire.

### 2.2 Instrumentation and Landing Analysis Protocol

**FIGURE 1:**
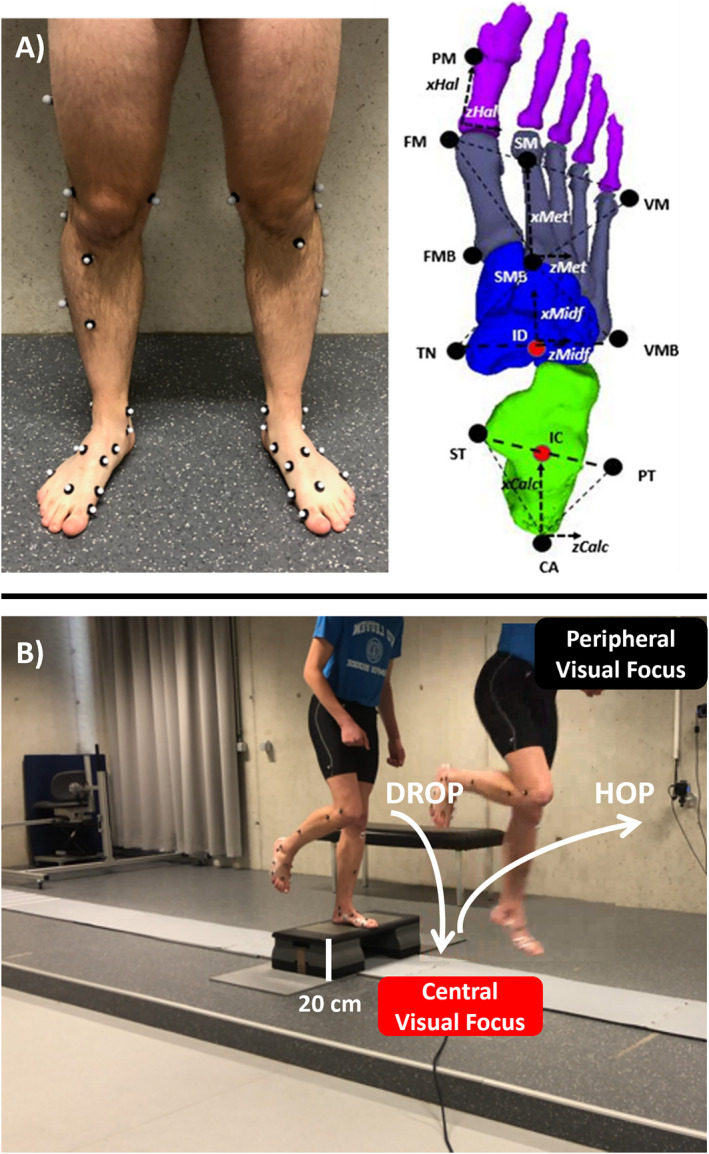
(A) Figure showing the marker placement with assumed rigid segments, anatomical landmarks and frames associated to the IOR‐4 Segment‐model used in the present study. Transverse planes (dash–dot triangles) and *X*‐ and *Z*‐axes (solid arrows) on these planes are shown. (B) Drop and hop with the red box representing the central visual focus and the black box representing the peripheral visual focus.

Tests were done at the Clinical Motion Analysis Laboratory of the University Hospitals Leuven. This lab was equipped with a 10‐m walkway surrounded by a passive optoelectronic motion analysis system (Vicon Motion System Ltd., Oxford Metrics) consisting of 12 infrared cameras. In the middle of the walkway, a force plate (Advanced Mechanical Technology Inc.) was integrated, with a pressure plate (Materialize Motion sensors, 2.8 sensors per cm^2^; Materialize Motion) placed on top. Data of the force and pressure plates were synchronized using the Materialize Motion 3D Box (Paal) and sampled at 200 Hz. All participants were instrumented according to the Rizzoli Foot Model (Figure 1) [24] and performed single‐leg drop and hop trials with their dominant limb. Each trial consisted of a forward drop from a 20‐cm box, followed by a maximal forward and upward hop. The task was performed in two distinct visual focus conditions. In the central focus condition, participants fixated their gaze directly on the landing spot throughout the movement. In the peripheral focus condition, participants kept their gaze straight ahead at a fixed visual target positioned at eye level (∼3 m in front of them). Compliance with visual focus instructions was monitored by the investigator. Participants performed 5 trials that were considered representative if participants did not deviate from the assigned focus condition and landed on the force plate when dropping of the box.

Data processing involved manual marker labeling and defining the individual landing cycles using the Nexus software (Vicon Motion System Ltd., Oxford Metrics, Oxford, UK). The contact phase was defined as the interval from initial contact with the force plate to push‐off. Processing of the kinematics associated with the Rizzoli Foot Model was performed using a BodyBuilder Plugin [12, 25]. This model considers the tibia as one rigid segment and the foot as a unit with four bony segments: calcaneus, midfoot, metatarsals, and hallux. The following inter‐segment angles (joints) were defined: ankle (including ankle and subtalar joint), Chopart, Lisfranc, and MTP‐1 joint. For these joints, the following joint centers were defined, respectively: (ankle) midpoint between medial and lateral malleoli; (Chopart) midpoint between the cuboid and the navicular bone; (Lisfranc) the base of the second metatarsal; (MTP‐1) the projection of hallux marker with halfway distance to the floor (Figure 1). An inverse dynamic approach was used to quantify the kinetics of the different joints which is the so‐called IOR‐4segment‐model1 [25, 26, 27]. All calculations were performed in an in‐house written Matlab code (Matlab 2022a, The MathWorks Inc., Natick, MA, US). All time‐dependent data (joint angle, moment and power waveforms) were subsequently normalized to 100% of the contact phase, according to the collected force and pressure data, and the mean pattern was calculated based on the recorded trials for each subject. The mean waveforms of internal joint moments and power were normalized by body mass before being averaged across subjects. In this study, we used the terms joint load to refer to internal joint moments, which represent the net muscular and passive contributions resisting external moments, and are commonly used as surrogates for joint loading [28] while energy refers to the capacity to perform mechanical work, represented by joint power, where positive values indicate generation and negative values indicate absorption. Although true joint contact forces require musculoskeletal modeling, internal moments and power offer clinically interpretable proxies relevant for a performance and rehabilitation context [28, 29, 30].

### 2.3 Data Analysis and Statistics

Joint kinematics, internal joint moments, and power generation/absorption for the four joints were presented as waveforms (dorsiflexion/plantarflexion, inversion/eversion, adduction/abduction) and analyzed over the entire contact phase. Statistical Parametric Mapping (SPM) (SPM1D version M.0.4.10, www.spm1d.org) was used to analyze time‐normalized landing phase profiles. This approach allows for a more comprehensive assessment of temporal variations in joint kinetics and kinematics, while avoiding statistical issues related to multiple comparisons across time‐series data. SPM achieves this by calculating a test statistic profile across each time point and modeling the behavior of random time‐dependent signals with a similar smoothness to the recorded data [31]. Additionally, we investigated zero‐dimensional kinetic outcome parameters of the four foot joints, which included the sagittal plane peak internal plantarflexion moment (Nm/kg) and peak power generation and peak power absorption (W/kg). Data were analyzed in SPSS (IBM Corp. Released 2023. IBM SPSS Statistics for Windows, Version 29.0.2.0 Armonk, NY: IBM Corp). We used an independent *t*‐test to compare the peripheral visual focus group to historical heel‐strike running data and the central visual focus group to heel‐strike running [12]. *p*‐values < 0.05 were considered statistically significant. Effect sizes (Cohen's d) for the differences in foot kinetics were calculated between groups to test the clinically meaningfulness of results. Criteria used were: small (0.2–0.5), medium (> 0.5 and ≤ 0.8), and large (> 0.8). The 95% confidence intervals (95% CI) are presented in the result section [32].

## 3 Results

**FIGURE 2:**
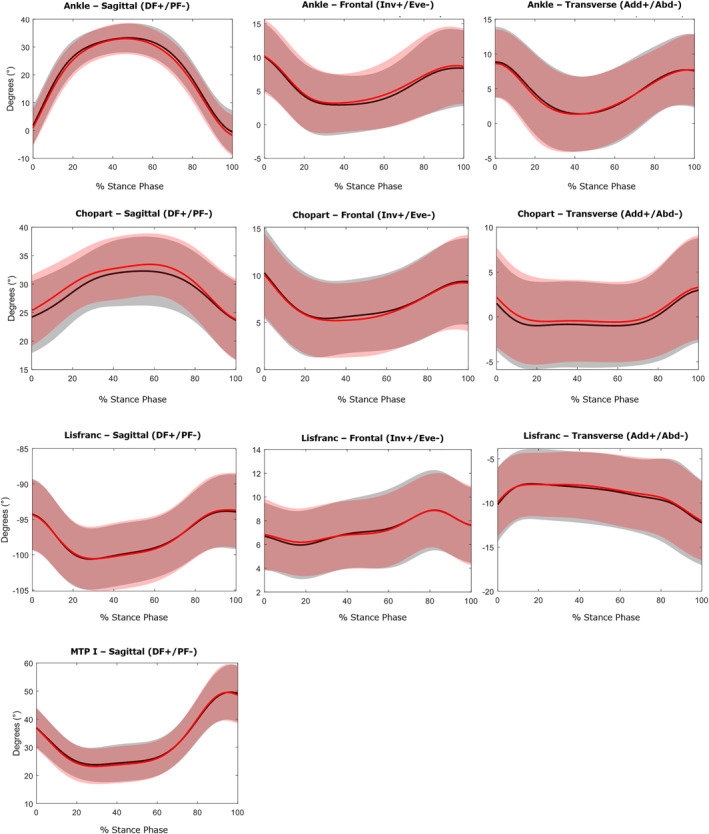
Joint kinematics in three planes: Sagittal (DF = dorsiflexion/PF = plantar flexion), Frontal (Inv = inversion/Eve = eversion) and Transverse (Add = adduction/Abd = abduction). Data of the peripheral visual focus are represented by a red line. Data of the central visual focus are shown with a black line.

**FIGURE 3:**
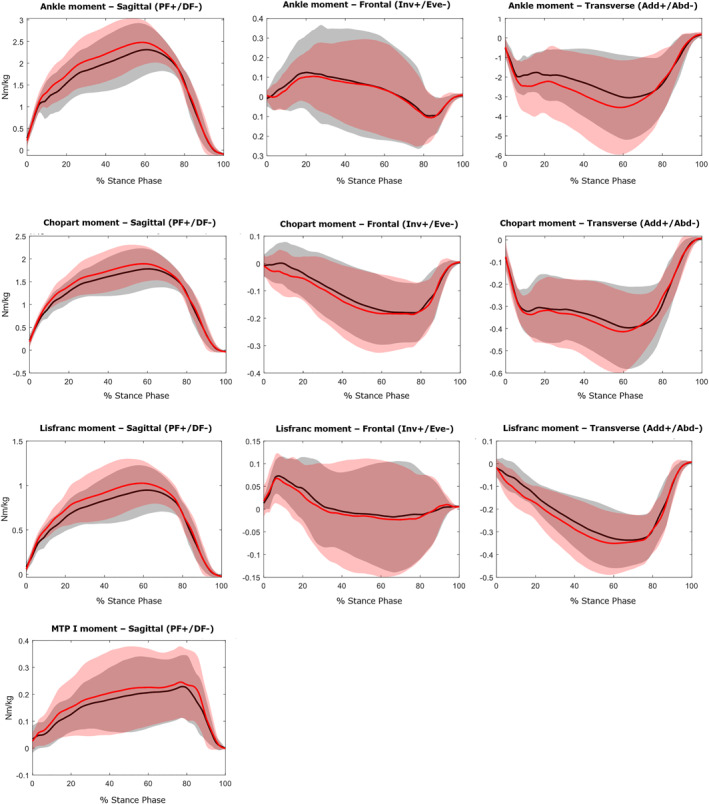
Internal joint moments in three planes: Sagittal (DF = dorsiflexion/PF = plantar flexion), Frontal (Inv = inversion/Eve = eversion) and Transverse (Add = adduction/Abd = abduction). Data of the peripheral visual focus are represented by a red line. Data of the central visual focus are shown by a black line.

**FIGURE 4:**
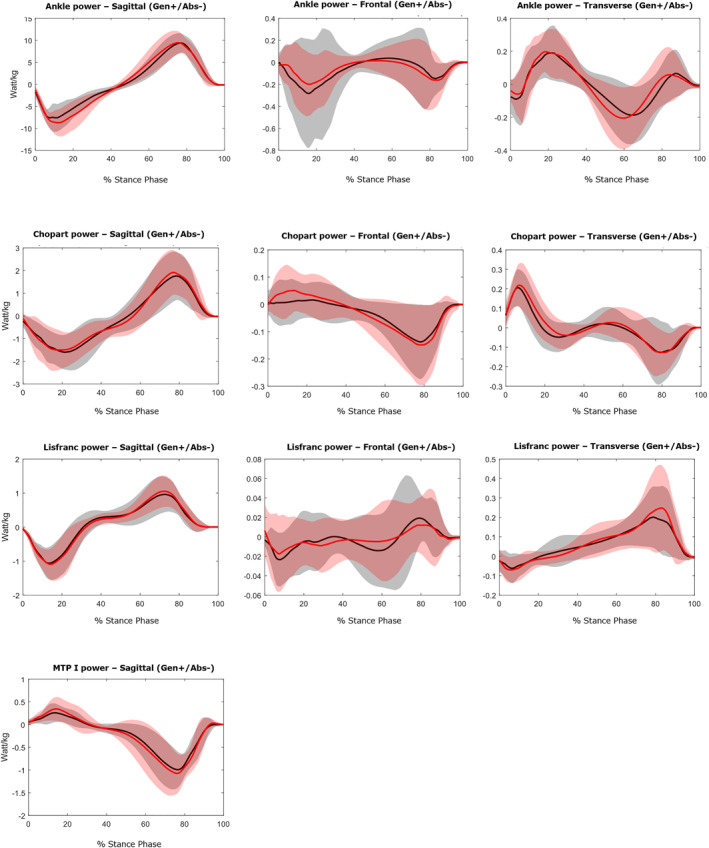
Joint powers in three planes: Sagittal (PF = plantar flexion/DF = dorsiflexion), Frontal (EV = eversion/Inv = inversion) and Transverse (Abd = abduction/Add = adduction). Data of the peripheral visual focus are represented by a red line. Data of the central visual focus are shown by a black line.

**FIGURE 5:**
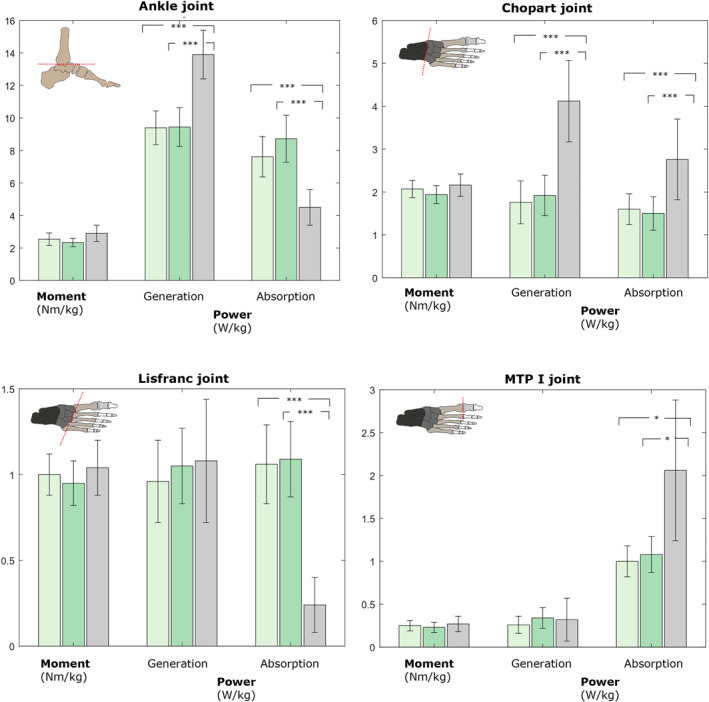
Bar chart visualizing the internal joint moments and power in the sagittal plane, comparing both single leg drop and hop tasks (light green: peripheral visual focus and dark green: central visual focus) with heel‐strike running (gray). *p < 0.05, **p < 0.01, ***p < 0.001.

Waveform data are presented in Figures 2, 3, 4. No significant differences were observed in the kinematic and kinetic variables between the two visual focus conditions for any of the analyzed foot joints. Table 2 and Figure 5 provide the zero‐dimensional kinetic parameters in the sagittal plane when comparing both single leg drop and hop conditions with heel‐strike running. Internal joint moments show no statistical difference (*p* > 0.05) between both drop and hop conditions and heel‐strike running, indicating similar joint loading. The peak power generation during heel‐strike running is significantly higher at the ankle (13.9 W/kg; *p* < 0.001) and Chopart (4.12 W/kg; *p* < 0.001) joint. The peak power absorption was significantly lower at the ankle (4.50 W/kg; *p* < 0.001) and Lisfranc (0.24 W/kg; *p* < 0.001) joint and higher at the Chopart (2.76 W/kg; *p* < 0.001) and MTP‐1 (2.06 W/kg; *p* < 0.05) joint during heel‐strike running.

**TABLE 2:** Comparison of internal joint moments (Nm/kg) and power‐related (W/kg) variables in the sagittal plane between the single leg drop and hop and heel‐strike running (Deschamps 2020).

	Mean [95% CI]			Mean difference		Cohen's *d* [95% CI]	
Variable	Peripheral focus	Central focus	Heel‐strike running	Peripheral versus. heel‐strike	Central versus. heel‐strike	Peripheral versus. heel‐strike	Central versus. heel‐strike
Ankle peak PF moment	2.54 [0.38]	2.33 [0.26]	2.90 [0.50]	0.21	0.57	0.75 [0.90]	0.42 [0.89]
Chopart peak PF moment	2.07 [0.20]	1.94 [0.21]	2.16 [0.26]	0.09	0.22	0.72 [0.90]	0.42 [0.87]
Lisfranc peak PF moment	1.00 [0.12]	0.95 [0.13]	1.04 [0.16]	0.05	0.09	0.21 [0.88]	0.11 [0.88]
MTP peak PF moment	0.25 [0.06]	0.23 [0.06]	0.27 [0.09]	0.02	0.04	0.01 [0.88]	0.19 [0.88]
Ankle peak power generation	9.39 [1.04]	9.44 [1.19]	13.9 [1.50]	4.51***	4.46***	2.05 [1.1]	1.77 [1.01]
Ankle peak power absorption	7.61 [1.24]	8.72 [1.44]	4.50 [1.10]	3.11***	4.22***	2.13 [1.06]	2.34 [1.10]
Chopart peak power generation	1.76 [0.50]	1.92 [0.47]	4.12 [0.95]	2.36***	2.2***	2.04 [1.05]	2.03 [1.05]
Chopart peak power absorption	1.60 [0.36]	1.50 [0.39]	2.76 [0.94]	1.16***	1.26***	1.07 [0.93]	0.88 [0.92]
Lisfranc peak power generation	0.96 [0.24]	1.05 [0.22]	1.08 [0.36]	0.12	0.03	0.07 [0.73]	0.09 [0.88]
Lisfranc peak power absorption	1.06 [0.23]	1.09 [0.22]	0.24 [0.16]	0.82***	0.85***	2.10 [1.06]	2.34 [1.10]
MTP‐1 peak power generation	0.26 [0.10]	0.34 [0.12]	0.32 [0.25]	0.06	0.02	0.10 [0.88]	0.19 [0.88]
MTP‐1 peak power absorption	1.00 [0.18]	1.08 [0.21]	2.06 [0.82]	1.06*	0.98*	1.74 [1.01]	1.39 [0.96]

Abbreviations: CI = confidence interval, PF = plantar flexion.

* *p* < 0.05.

**p < 0.01.

*** *p* < 0.001.

## 4 Discussion

The primary objective of this cross‐sectional study was to characterize the kinematic and kinetic profiles of the ankle, Chopart, Lisfranc, and MTP‐1 joints during a single‐leg drop and hop task under two visual focus conditions. This study provides for the first time documentation of joint loading across specific foot joints during a dynamic, single‐leg task.

No significant differences in biomechanical variables were observed between a central visual focus and a peripheral visual focus despite our expectation that the latter might alter joint loading. Our findings contrast with prior monosegmental research showing that altered visual focus has effects on the kinematics of the ankle joint. Ko et al. (2022) [20] reported increased ankle inversion under peripheral gaze conditions, while Terada and Gribble (2015) [21] observed reduced eversion moments at initial contact. However, it is known that monosegment foot models lead to overestimation of both ankle joint kinematics and kinetics, making comparison to aforementioned authors difficult [33, 34]. Furthermore, the lack of significant differences in foot joint kinematics and kinetics suggests that neuromuscular control and proprioceptive feedback, rather than visual input, are the primary drivers. This aligns with results from Lieberman and Hoffman (2005), who found that optic flow during landing does not significantly influence preparatory muscle activation during self‐initiated falls. Additionally, the high density of cutaneous receptors in the foot sole may have played a critical role. Cutaneous afferent receptors in the plantar surface of the foot provide essential feedback for the regulation of balance and gait mechanics [35]. This dominant sensory input may override the influence of visual focus. The importance of sensory feedback from the foot's cutaneous receptors is underscored by Eils et al. (2002), who demonstrated that reducing plantar sensation significantly alters pressure distribution during gait [36].

The landing height was set at 20 cm to ensure task feasibility and reduce injury risk in asymptomatic individuals, while still representing a commonly used height in rehabilitation. Previous studies have indicated that landing from heights below 30 cm may not always reach the threshold required to significantly alter lower extremity sagittal plane biomechanics, with less conclusive results in the frontal plane [37]. Given these considerations, we hypothesize that two key factors may explain the lack of significant differences between visual focus conditions. First, as this study involved healthy individuals with intact proprioception, any reduction in visual input may have been compensated for by proprioceptive mechanisms [38]. Prior research indicates that proprioception plays a dominant role in lower limb motor control, particularly during controlled landing tasks. Second, the need for precise visual information may be lower for predictable, structured tasks such as a fixed‐height landing in a laboratory setting, compared to more dynamic and unpredictable real world movements. During controlled tasks, proprioceptive cues can often provide sufficient feedback for motor control, reducing reliance on visual input [39]. Additionally, the visual discrepancy between central and peripheral focus may have been minimal in this context, whereas in real world environments with more complex visual stimuli, such differences could have a greater impact on movement execution [40].

The second objective of this study was to contextualize results of foot joint biomechanics during single‐leg drop and hop using heel‐strike running data. Peak plantarflexion moments at the ankle joint were similar between the single‐leg drop and hop and heel strike running. Similar patterns were found for plantarflexion moments at the level of the Chopart, Lisfranc and MTP‐1 joint. These comparable joint moments suggest that joint loading during these activities are similar, with joint moments used as surrogate for joint loading [29, 30]. In the absence of musculoskeletal modeling, this approach provides a practical method for estimating joint loading. However, this surrogate or proxy approach is primarily evidenced at the level of the knee joint. For other lower limb joints, and especially the joints of the multisegment foot, such relationships have not been established. The biomechanical complexity of the foot, with multiple small articulations and load‐sharing across segments, makes it challenging to infer actual joint loading from inverse dynamics alone.

Despite the similarities in joint moments, peak power generation at the ankle and Chopart joint was significantly lower (*p* < 0.001) during the single leg drop and hop than during heel‐strike running. The higher power generation in heel‐strike running typically supports the dynamic nature of running, which involves continuous propulsion and forward momentum. The foot muscles generate more power to maintain speed, whereas the single‐leg drop and hop is more focused on controlled landing in order to hop forward. Moreover, elastic recoil of the Achilles tendon, foot muscles and plantar aponeurosis further add to the power generation at the ankle and Chopart joint [41, 42, 43].

Power absorption shows notable differences between both activities. For the MTP‐1 joint, peak power absorption during heel‐strike running is significantly higher (*p* < 0.05) compared to the drop and hop. During running, the MTP‐1 joint plays a crucial role in stabilizing the foot and absorbing impact forces during the stance phase. As the foot strikes the ground and transitions to push‐off, the MTP‐1 joint must absorb significant forces to control the forward motion and prepare for the next stride. This requires substantial energy dissipation. In contrast, the drop jump primarily emphasizes controlled landing. Although the MTP‐1 joint still absorbs impact forces, the overall demand for energy dissipation may be lower because the focus is on stabilizing the body and preparing for the next movement, rather than continuous propulsion.

Another difference between activities lies in the distribution of power absorption and generation across the foot joints. During the single‐leg drop and hop, the ankle, Chopart, and Lisfranc joints exhibit a balanced ratio, with power absorption nearly equating power generation. This suggests a coordinated effort among these joints to effectively dissipate impact forces and maintain stability during landing. Intriguingly, this balanced ratio between absorption and generation is not true for the MTP‐1 joint. This joint stands out as a significant site of power absorption during the single‐leg drop and hop, potentially serving as a pivotal point for force attenuation during foot‐ground contact and push‐off. In contrast, during heel strike running, the ankle, Chopart, and MTP‐1 joints predominantly function as power absorbers, facilitating shock absorption upon initial heel‐ground contact with the lisfranc joint functioning as a primary power generator, likely contributing to propulsion and forward momentum of body mass.

The differences in power generation and absorption highlight the unique mechanical demands of each activity. Single‐leg drop and hops require controlled landing mechanics, focusing on balance and stability, in preparation for the hop phase that follows. In contrast, heel‐strike running demands continuous propulsion and effective impact absorption. Understanding these biomechanical differences is crucial for tailoring training and rehabilitation programs. For example, exercises that enhance power generation may support individuals aiming to improve forward propulsion during activities such as walking or jogging. Conversely, exercises focusing on impact absorption and stability might be more relevant for patients engaged in tasks involving repetitive stepping, jumping or landing.

This study provides a deeper insight in foot joint biomechanics during different tasks, however there are also limitations that should be carefully considered to avoid generalizability when interpreting task‐related differences in joint kinetics. First, the male dominance in our study limits the generalizability of these findings, particularly in light of established sex differences in landing mechanics and injury risk. Females, for example, often exhibit greater knee valgus and different muscle activation patterns during landing [44], which may potentially affect joint moments and power generation at the foot. Future studies should aim for a more balanced and larger cohort to capture these differences and ensure broader applicability of the results. Second, while the use of previously published heel‐strike running data (Deschamps et al., 2020) [12] provides valuable biomechanical context, this comparison introduces important limitations. The running cohort differed in sex composition (mixed vs. all male), and although demographic variables such as age, height, and BMI were similar, no formal statistical comparisons were made. Additionally, methodological constraints differed between the two tasks: visual focus and landing mechanics were tightly controlled in our study, while these factors were uncontrolled in the historical data. It is also likely that foot strike patterns differed (forefoot strike during drop‐hop vs. heel strike during running), which may independently influence joint kinetics. Finally, the running dataset had a relatively small sample size (*n* = 7), further limiting statistical robustness. As such, this analysis should be interpreted as a contextual comparison rather than a formal between‐group inference. Future studies should aim to collect both task conditions within the same cohort under standardized conditions to enable stronger, within‐subject comparisons.

## 5 Conclusion

This study provides the first insights into foot joint kinematics and kinetics during single‐leg drop and hop tasks under different visual focus conditions, while also contextualizing them with heel‐strike running. Our findings suggest that visual focus, central versus peripheral, has minimal impact on foot joint biomechanics in non‐injured individuals, likely due to dominant proprioceptive control. Future research should examine whether individuals with pain or proprioceptive deficits rely more on visual feedback.

Although joint moments were comparable across tasks, power demands differed, with running emphasizing propulsion and single‐leg tasks prioritizing impact absorption and stability. These findings support single‐leg landing exercises as controlled alternatives to running for rehabilitation but highlight their limitations in replicating propulsion demands. Future research should explore biomechanical variations in symptomatic populations, elite athletes, and other dynamic tasks such as cutting, pivoting, squatting, and lunging to expand our understanding of foot joint biomechanics in functional movement and rehabilitation.

## Author Contributions

**Nicolas Haelewijn:** conceptualization, methodology, software, validation, formal analysis, investigation, writing – original draft, writing – review and editing, visualization, project administration. **Filip Staes:** methodology, resources, data curation, writing – original draft, writing – review and editing, visualization, supervision. **Evie Vereecke:** methodology, resources, data curation, writing – original draft, writing – review and editing, visualization, supervision. **Kevin Deschamps:** conceptualization, methodology, software, validation, formal analysis, investigation, resources, data curation, writing – original draft, writing – review and editing, visualization, project administration. **Stijn Rosseel:** methodology, writing – original draft, writing – review and editing, visualization.

## Ethics Statement

The authors are accountable for all aspects of the work in ensuring that questions related to the accuracy or integrity of any part of the work are appropriately investigated and resolved. The study was conducted in accordance with the Declaration of Helsinki (as revised in 2013). Each participant signed the informed consent form. This study was approved by the Ethical Committee UZ/KU Leuven (MP019006).

## Conflicts of Interest

The authors declare no conflicts of interest.

## Data Availability

The data that support the findings of this study are available from the corresponding author upon reasonable request.
